# Preserved Ophthalmic Anti-Allergy Medication in Cumulatively Increasing Risk Factors of Corneal Ectasia

**DOI:** 10.3390/biology12071036

**Published:** 2023-07-22

**Authors:** Tom Paterson, Serap Azizoglu, Moneisha Gokhale, Madeline Chambers, Cenk Suphioglu

**Affiliations:** 1 NeuroAllergy Research Laboratory (NARL), School of Life and Environmental Sciences (LES), Faculty of Science, Engineering and Built Environment (SEBE), Deakin University, 75 Pigdons Road, Geelong, VIC 3216, Australia; tpaterson@deakin.edu.au (T.P.); serap.azizoglu@deakin.edu.au (S.A.); moneisha.gokhale@deakin.edu.au (M.G.); mchambers@deakin.edu.au (M.C.); 2 Deakin Optometry, School of Medicine, Faculty of Health, Deakin University, 75 Pigdons Road, Geelong, VIC 3216, Australia

**Keywords:** ocular allergy, cornea, keratoconus, topical ophthalmic medication, benzalkonium chloride, antihistamine, mast cell stabilisers

## Abstract

**Simple Summary:**

Allergy is a global health issue, and with the advent of modern medicine comes widely available treatments for allergy symptoms. For the localised treatment of itchiness and inflammation associated with ocular allergies, those affected typically use topical anti-allergy eyedrops. This review sought out previous research to investigate the prevalence of ocular allergy, the contents of anti-allergy medication and the pathophysiology of corneal thinning. We found that benzalkonium chloride, the most common preservative for multiuse eyedroppers, has a documented effect on corneal cell viability, weakening the corneal structure. When compounded by common risk factors for corneal thinning, such as the release of inflammatory enzymes and mechanical pressure applied from rubbing your itching eyes, the cellular damage benzalkonium chloride may inflict on the cornea may further increase the risk of permanent corneal damage.

**Abstract:**

The prevalence of allergies is rising every year. For those who suffer from it, ocular inflammation and irritation can be inconvenient and unpleasant. Anti-allergy eyedrops are a readily available treatment for symptoms of ocular allergy (OA) and can help allergy sufferers regain normal function. However, the eye is a delicate organ, and multiuse eyedrops often utilise preservatives to deter microbial growth. Preservatives such as benzalkonium chloride (BAK) have been shown to induce decreased cell viability. Therefore, during a period of high localised inflammation and eye rubbing, it is important that the preservatives used in topical medicines do not contribute to the weakening of the corneal structure. This review explores ocular allergy and the thinning and protrusion of the cornea that is characteristic of the disease keratoconus (KC) and how it relates to a weakened corneal structure. It also describes the use of BAK and its documented effects on the integrity of the cornea. It was found that atopy and eye rubbing are significant risk factors for KC, and BAK can severely decrease the integrity of the corneal structure when compared to other preservatives and preservative-free alternatives.

## 1. Introduction

The worldwide incidence of allergies has shown a consistent rise. A study by Wilson et al. [1] showed an increase in asthma prevalence from 7.5% of the surveyed population to 12.2% between the years 1990 and 2003 in South Australian households, demonstrating a 63% increase in prevalence. Likewise, De Marco et al. [2] showed an increase in allergic rhinitis prevalence in Italy from 16.8% to 25.8% between the years 1991 to 2010, which is a 34% increase over the course of nearly two decades. While a difference in prevalence can be attributed to multiple and often differing factors, the density of reported individuals presenting symptoms of hypersensitive immune responses has increased with each year. Despite consistent industry research into anti-allergy treatments, the majority of results have only yielded allergy management strategies [3]—medicines and lifestyle changes that, while able to improve the individual’s quality of life, serve only to provide momentary relief from allergy symptoms.

Generally, allergens will garner a hypersensitivity response by binding to antigen-presenting cells and initiating an IgE-mediated allergenic response [4], resulting in common symptoms such as itchiness, sneezing and runny nose. Depending on the type of allergy and allergen, the symptoms of inflammation can vary, leading to the need for a distinction between the types of allergies. Among the population suffering from allergy in one form or another, it is believed that up to 40% of those with allergy experience symptoms localised to the eye, classified as ocular allergy (OA) [5]. The typical symptoms of OA include itchy eyes, watery eyes and photosensitivity [6]. These symptoms can make it very uncomfortable for those who suffer from OA, leading to a need to seek relief. However, instinctively rubbing the eyes can put mechanical pressure on them, which has been shown in previous studies to be a risk factor for thinning and deformation of the cornea [7]. For more effective relief, patients may turn to self-medicating with anti-allergy eyedrops [6], which are medicated solutions applied topically to the surface of the eye. Antihistamines, mast cell stabilisers, combination drops and corticosteroids are all commercially available topical anti-allergy drops. Each treatment is available over the counter without the need for a prescription, with the exception of corticosteroids and olopatadine [8]. A common preservative used in multiuse bottles of ophthalmic drops is benzalkonium chloride (BAK), a powerful quaternary ammonium that discourages microbial growth and has previously been shown to have adverse effects on corneal epithelial viability [9]. Allergies can occur seasonally or year-round [10], and long-term use of eyedrops over several months can increase the likelihood of adverse effects [11]. Studies on the effects of ophthalmic medications have shown the dose-dependent cell death of corneal epithelial cells in both in vivo and in vitro contexts [12,13]. However, the consensus from such studies attributes decreased cell viability to the BAK preservative. Similarly, anti-allergy eyedrops with BAK have also shown a decrease in cell viability [14].

Allergy has previously been linked to corneal ectasia through both inflammatory mediators [15] and mechanical pressure applied through eye rubbing [7,16,17]. However, the unknown factor is whether certain properties in topical anti-allergy medicines are weakening the cornea via either weakening of cell junctions or induction of corneal cell death, which may be leaving the cornea susceptible to deformation during a period in which eye rubbing is at its most frequent. The present article aims to provide a comprehensive review of the cornea, the prevalence and burden of allergy, and the contents and previously observed effects of anti-allergy medications. The collection of information from prior research will suggest a reason to investigate the pathological corneal response to the long-term use of topical allergy treatments.

## 2. Definitions

### 2.1. Keratoconus

**Figure 1:**
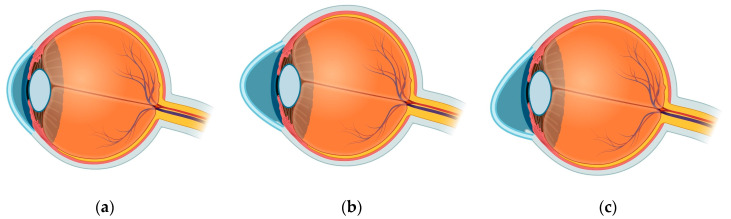
Depiction of the side profiles for the two forms of keratoconus: (**a**) A normal cornea for comparison. (**b**) The more common cone-shaped protrusion. (**c**) The less common ovoid cone [20]. Diagram constructed using BioRender.

Keratoconus (KC) is an ectatic disease characterised by the progressive thinning and protrusion of the cornea. The disease can express both bilaterally and symmetrically [18]. In most cases of advanced KC, the cornea will protrude into a conical shape, thinning towards the cornea’s peak [19]. KC is clinically categorised into two types: The more common conformal cone structure, as seen in Figure 1b, has the protrusion centred within a few millimetres of the visual axis with a minimal protrusion base diameter. Figure 1c depicts the less common sagging ovoid protrusion, the centre of the cone often sits further below the visual axis and has a larger base diameter; the ovoid form of KC can lead to more severe complications and can lead to a worse quality of life [20]. The name “keratoconus” was coined by Nottingham in 1854 when he first described the disease [21]. He derived keratoconus from the Greek words *kerato* (cornea) and *konos* (cone) describing the pathology of the condition. KC is a disease encompassing ectatic conditions in the cornea.

### 2.2. Ocular Allergy

Ocular allergy (OA) is the broad term representing multiple types of allergic conjunctivitis (AC), the hypersensitive immune response to otherwise harmless substances. Substances such as pollen, dust mites and animal dander are among the most common to induce mild to severe inflammatory reactions in affected people [5,22]. The clinical presentation of AC falls under four main categories: seasonal allergic conjunctivitis (SAC) and perennial allergic conjunctivitis (PAC), the two most common forms of OA accompany many of the milder signs and symptoms of ocular allergy such as itching, swelling and vascularisation [10]. The more chronic OA expressions, vernal keratoconjunctivitis (VKC) and atopic keratoconjunctivitis (AKC) accompany more intense itching sensations, swelling, mucous secretions and photophobia. Giant papillary conjunctivitis is an associated condition but not a “true ocular allergic reaction” as it is the result of repeated mechanical irritation often associated with and aggravated by OA [10].

## 3. Cornea Structure

### 3.1. Ocular Surface

**Figure 2:**
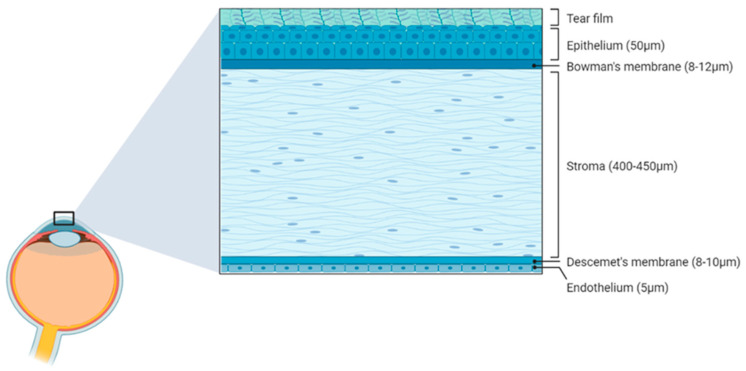
A diagram showing the 5 layers of the cornea and their approximate thickness. Diagram constructed using BioRender.

The external surface of the human eye consists of the conjunctiva and the cornea in a 17:1 ratio with a total surface area of approximately 1800 mm^2^ [23,24]. The human cornea, which is at the centre of the “visible” portion of the eye, acts as a transparent barrier between the environment and the intraocular contents. The cornea comprises an arrangement of five layers (Figure 2) specifically arranged to ensure the transparency of the cornea and the precise passage of light. The most anterior being the stratified squamous epithelial layer (50 µm), followed by a thin Bowman’s layer (8–12 µm); and at the centre, a highly organised stroma, which is the thickest (80% of corneal thickness) layer of the cornea (400–450 µm); the pre-Descemet’s membrane or Dua’s layer (6–15 µm); a thin Descemet’s membrane (8–10 µm) and, finally, a single-layered endothelium (5 µm) [25]. Each of the layers adhere tightly to the adjacent layer with anchoring proteins. Collagen networks provide support and rigidity within the layers and are organised precisely to retain the transparency required for the passage of light through to the anterior chamber. To allow for light to be perceived while also keeping the cornea relatively safe, a transparent layer called the tear film exists, blanketing the surface of the eye, shielding it from foreign particles and from drying out in the wind.

### 3.2. Tear Film

**Figure 3:**
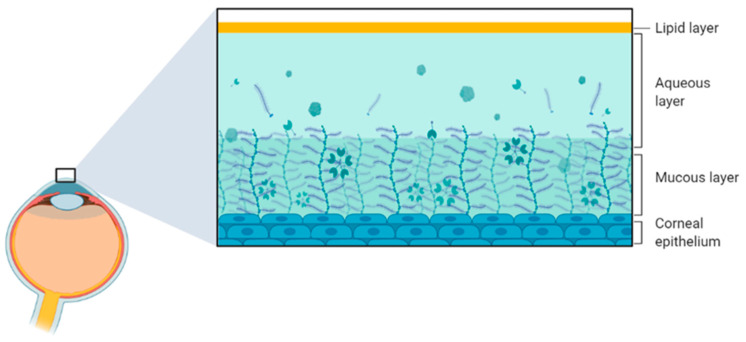
A diagram showing the three phases of the tear film, responsible for the protection and lubrication of the corneal epithelium. Diagram constructed using BioRender.

The tear film is a fluid layer at the anterior end of the corneal and conjunctival epithelium. This layer (Figure 3) acts as an additional barrier to entry that stops incoming allergens such as pollen, dust and other small irritants before they reach the surface of the eye, maintaining the crucial clarity and transparency. The constant drainage of the tears also ensures dilution of these allergens, making it more difficult for them to reach the corneal and conjunctival surface. Consisting of an exterior lipid layer, a middle aqueous layer and an interior mucous layer, the tear film is necessary for the lubrication and protection of the corneal epithelium [26]. Additionally, as the cornea is not vascularised, the tear film contains dissolved nutrients to deliver to the corneal layers [25]. Each layer of the tear film has a specific function. The lipid layer is responsible for protecting the tear film fluid from evaporation and pathogens. The aqueous layer is a solution of essential electrolytes, proteins and metabolic products. Further, the mucous layer is responsible for maintaining the fluids’ structure [27]. While previously defined as a layered structure, the tear film has been more recently redefined since it is more akin to a phased gradient of a concentrated solution that is consistently flowing and refreshing itself [28]. Furthermore, the precise composition of tears at any given time varies and is dependent on the current health of the ocular surface.

The outermost layer of the cornea is the corneal epithelium, a layer of non-keratinised stratified squamous epithelial cells [25], which acts as a primary barrier to incoming particles. The corneal epithelium accounts for 10% of the corneal thickness, while the stroma accounts for approximately 80–90% of corneal thickness [29]. The stroma is considered the largest contributor to corneal stability and is, therefore, more important to the structural integrity of the cornea. The stroma’s structural importance can be attributed to the composition of its extracellular matrix consisting mostly of a highly organised collagen network interwoven into lamella [25]. The thinning of the corneal stroma, otherwise known as corneal ectasia, has been associated with a decrease in epithelial density, a worrying association that has implications on the form and function of the cornea.

## 4. Ocular Allergy

### 4.1. Allergic Conjunctivitis

OA is a form of localised allergic response that causes inflammation in the eye due to contact with allergens. It is believed that up to 40% of allergic individuals suffer from some form of OA [5]. Cases of AC can be divided into four distinct categories as described below.

#### 4.1.1. Seasonal and Perennial Allergic Conjunctivitis

SAC and PAC are the most common forms of allergic conjunctivitis. The symptoms of both conditions are similar; it is common for these forms of AC to induce an itching sensation (88%), tearing (88%), swelling (72%) and vascularisation (78%). The distinction between SAC and PAC is attributed to the allergens to which the individual is hypersensitive. SAC is associated with seasonal allergens such as pollen, an allergen that is in high airborne concentration during the months of summer and spring, and PAC is associated with more permanent year-round allergen exposures such as dust mites, mould or animal dander [22,30]. As a result of the large variety of allergens that can trigger SAC and PAC, large populations of those who experience OA, experience both types of AC resulting in prolonged periods of time during which the individual’s eyes and corneas are inflamed and at risk of damage.

#### 4.1.2. Vernal Keratoconjunctivitis

VKC is a more chronic form of OA than SAC or PAC and typically affects younger people, particularly younger males. It is bilateral and typically accompanies a more severe itching sensation, photophobia and mucus discharge, typically followed by sticking eyelids during the morning [31].

#### 4.1.3. Atopic Keratoconjunctivitis

AKC is the most potentially damaging form of AC, as it can lead to superficial punctate keratopathy and eventually potential blindness due to its characteristic severe inflammatory response [32]. Alongside the common itching and redness, AKC can present symptoms in the form of pain, chronic swelling and blurred vision. AKC is often present more in males aged 30–50 years and occurs alongside atopic dermatitis [10].

### 4.2. Pathophysiology

The immunoglobulin E (IgE)-mediated immune response is induced by exposure to allergens along the ocular surface and their subsequent complex formation with IgE receptors displayed on the surface of mast cells [33,34]. The deposition of allergens on the conjunctival mucosa is processed by antigen-presenting cells along the epithelium, which then present the processed antigens to naïve Th0 cells’ major histocompatibility complex class II molecules. Among genetically predisposed allergic individuals, the Th0 cell differentiates into Th2 lymphocytes and releases cytokines such as IL-4, 5 and 13, which stimulate IgE production by B cells [4]. IgE then contacts the local mast cells causing them to degranulate and thereby releasing inflammatory mediators such as histamines, tryptase, leukotrienes and prostaglandins, which go on to instigate the allergic response in cells. This process can result in ocular itching [35]. Upon re-exposure to allergens, the antigens complex with complementary mast-cell-bound IgE antibodies, which are secured on mast cells via their receptors, and the cross-linking of such IgE receptors signals for mast cell degranulation [36].

### 4.3. Burden of Allergy

According to the literature [2,37], the prevalence of allergies has been rising for many years. Susceptibility to AC can persist throughout a patient’s lifespan and can severely impact a person’s quality of life. The most common allergens such as pollen and dust, can be found anywhere and can often be hard to avoid in daily life. Allergy symptoms can place a significant burden on lifestyle and life choices, affecting not only individuals but also their families and the economy due to the debilitated state that allergy symptoms place on working citizens. A quality-of-life survey conducted by Smith et al. [38] in 2002 found that individuals who suffer from SAC have an overall 15% decrease in work productivity while suffering from allergy symptoms. This loss of productivity has the potential to result in decreased productivity and wages. This is concerning when it is considered that individuals affected by allergies and their families were responsible for 49% of the total $7.8 billion economic cost of allergies, or 86% if wellbeing was considered. In Australia, a study surveying atopic disease in Melbourne schools between 1993 and 2002 showed a 3% increase in rhinitis prevalence [39]. Melbourne, Australia, has observed the most disastrous events related to reactions with seasonal grass pollen such as the infamous thunderstorm asthma outbreak event of 21st of November 2016. This incident in metropolitan Melbourne resulted in the hospitalisation of thousands as well as 10 associated deaths [40]. The evaluation of patient medical data showed that as many as 88% of the patients admitted during the event had a history of rhinitis [41]. In the wake of events such as these, focus will need to be placed on further investigating the interactions and subsequent over-reactions between allergenic particles and the immune system.

The symptoms of OA can range from mild irritation to severe pain. Itchy and swollen eyes with the potential for foreign body sensations will lead patients seeking instant relief to rub their eyes. This is a natural response, which is hard to combat [42]. While each incident of eye rubbing is less than a few seconds in duration, eye rubbing is a common occurrence that can come about instinctively several times a day. The frequency of eye rubbing is exacerbated by irritants and inflammation; this may be a cause for concern as excessive mechanical stress has been previously linked to ocular ectasia, the deformation of the cornea [7,16]. In one study conducted by Shneor et al., 2013 [43], it was observed that 65% of patients with a known keratoconus diagnosis had a history of eye rubbing. It has also been shown that eye rubbing can induce the expression of pro-inflammatory cytokines and proteases [42]. Consequently, symptoms of OA can lead to the acquisition of self-prescribed topical medication, easily accessible anti-allergy eyedrops that contain among other things the anti-allergy active ingredient, sometimes in combination with a vasoconstrictor, and if in a multiuse container, a preservative.

## 5. Topical Treatments

Topical ophthalmic medicines such as antihistamines and mast cell stabilisers are often privately sought-after and self-administered in mild/severe cases of OA to help alleviate irritating symptoms, yet there has been little research into the effects of these solutions on corneal cell integrity. Research found that, among patients suffering from allergies in different forms, OA patients will be more likely to actively take medication for their symptoms [44]. The majority of anti-allergy eyedrops are available over the counter with only a few requiring prescription. A questionnaire conducted with a sample of first-time patients at an ophthalmology clinic asked patients how they had treated their ocular illness prior to their visit. The questionnaire revealed that 25.6% of the patients reported self-medicating prior to seeking professional advice and 24.3% had medicine prescribed by a general physician [8]. Of the patients who self-medicated, only 31% followed advice from a pharmacist while 25% had purchased eyedrops at their own discretion, and 24% followed suggestions from friends and family. Additionally, 14% of the total patient sample stated that they did not remember the name of the medication they were using, and only 3% were aware of its potential side effects [8]. With such a significant proportion of the population uneducated about the contents of their medication, the components that comprise the medication must entail the lowest risk of adverse effects. In the current Australian market, anti-allergy eyedrops are sold as antihistamines, mast cell stabilisers or combination drops that target both; most contain benzalkonium chloride (BAK) as a preservative. With the exception of pheniramine maleate, ketotifen is the only commercial selection with both a preserved and non-preserved option.

Antihistamine active ingredients, such as levocabastine and pheniramine maleate (Table 1), act upon H1 histamine receptors as antagonists, specifically targeting the histamine signalling pathway rather than mast cell degranulation [45,46]. Pheniramine maleate antihistamine solution is commercially mixed with the vasoconstrictor naphazoline hydrochloride to assist in reducing vascularisation in the sclera [47]. Mast cell stabilisers, such as sodium cromoglycate inhibit the degranulation of mast cells preventing the excessive release of chemical mediators, including histamines into the extracellular environment. Further, combination agents such as ketotifen and olopatadine are active ingredients that can be considered to have dual action by inhibiting H1 receptors and stabilising mast cells as a mode of action [47,48]. Antihistamines and mast cell stabilisers are recommended to be used as long as symptoms of allergies may persist. Mast cell stabilisers in particular have been observed to have a delayed onset of action and are recommended to be taken 2 weeks prior to expected exposure to allergens for maximised effectiveness [47].

The much more chronic symptoms of VKC and AKC cannot be treated with antihistamines and mast cell stabilisers alone; these conditions require corticosteroids for their immunosuppression and anti-proliferation properties. Topical corticosteroids such as fluorometholone and dexamethasone work by hindering transcription of Th2-derived cytokine genes, thereby limiting the differentiation of T-lymphocytes into Th2 lymphocytes [34]. Corticosteroids require the approval of a specialist before treatment and are only ever approved for short treatment durations of less than 2 weeks; this is due to the medicine’s effects of delaying wound healing and raising intraocular pressure [49].

The human tear volume under normal conditions has been measured to be around 7–9 µL to achieve a full coat along the eye surface, refreshing at a rate of up to 2.2 µL/min [24]. Average commercial eyedrops dispense approximately 30–50 µL of ophthalmic solution to the cornea depending on the proprietary dispenser; despite the large increase in volume from the eyedrop application, little of the active ingredient actually penetrates the cornea. When dispensed onto the open eye, the first barrier to entry is reflex blinking; the large increase in volume from a single drop into the eye activates drainage systems and reflex blinking, leaving only 7 µL of solution remaining after initial drainage [50]. After the initial decrease in volume due to blinking, the tear film and tear replacement impose a second barrier to entry; the lipid phase and the aqueous phase can repel both hydrophilic and hydrophobic drugs, respectively. The aqueous phase also contains many proteins and metabolic products that can inactivate drugs, making drug penetration difficult to achieve. The corneal epithelium is yet another barrier of tightly adherent lipophilic tissue, which favours the passage of hydrophobic drugs more than hydrophilic ones [24]. It is estimated that these factors can eliminate as much as 60% of the active ingredient after only two minutes, with the active ingredient diluted to 1/1000 of its original concentration after 8 min, and after 15 min, the drug is almost entirely eliminated. During this time, the active ingredient is absorbed through the cornea and sclera, with as little as 5% of the original dose reaching the aqueous humour in the anterior chamber [51]. Due to the factors listed above, replication of such conditions in vitro would require a modified volume and concentration.

### Benzalkonium Chloride

Anti-allergy eyedrops are often purchased in multiuse bottles, intended to treat symptoms over long periods of time. To prevent the proliferation of microbes within the solution, preservatives must be used as antimicrobial agents. One antimicrobial agent that is most common in commercially prepared solutions is benzalkonium chloride (BAK), a quaternary ammonium molecule that possesses hydrophilic and hydrophobic qualities that are useful to stabilise insoluble drugs, enhancing penetration [52]. BAK has shown considerable efficacy against many pathogens; however, it has also been linked to multiple detrimental effects on the tear film and corneal surface [14,52]. The literature has demonstrated that 0.02% BAK can increase the permeability of the cornea and cause cell death within 15 min of application in vivo [12]. Pauly et al., in 2012, demonstrated that, at 0.01%, BAK induced CD45 expression, apoptosis and general disorganisation of the corneal epithelium [11]. In another in vivo study, 0.01% BAK was shown to expedite the formation of dry spots in the tear film up to twice as fast as in the control group of human volunteers [53]. Dry spots can cause irritation, burning eyes and corneal irregularities leading to reduced vision quality. Furthermore, BAK, being a cationic compound, has the ability to interact with mitochondrial function, and in work conducted by Datta et al., in 2017, it was observed that 0.1% BAK caused an inhibition of mitochondrial function in corneal epithelial cells. At half the concentration of BAK, Vitoux et al. [9] found results of corneal epithelial apoptosis, oxidative stress and morphological alterations in vitro.

Researchers studying the effects of BAK on the corneal epithelium often test its use in commercial treatment where both the active ingredient and a preservative are present. They do this to also determine what other factors may be attributed to cytotoxicity in practice. While many commercially available treatments are only available preserved, ketotifen fumarate is most commonly available preserved with BAK but is also available as a preservative-free option, foregoing the multiuse bottle format for sterile, single-use, sealed droppers. Guzman et al. [14] demonstrated a dramatic gap in cell viability after exposing a stratified corneal epithelial cell culture to a 10% dilution of commercial ketotifen both preserved and non-preserved. The non-preserved ketotifen caused no significant corneal cell viability drop compared to the control after a 24 h period. In contrast, the BAK containing solution reduced cell viability to approximately 70% within just 20 min of exposure; this decreased further to around 40% viability after the 24 h treatment period was complete.

As for the corticosteroids, while they have documented detrimental effects on the rate of wound healing in the long term, a study by Kim et al., 2016 [54] demonstrated that fluorometholone preserved with BAK significantly decreased the cell viability of corneal cells in vitro, demonstrating cell shrinkage and loss of adhesion. Comparatively, they concluded that fluorometholone independent of BAK had better immunosuppressant properties than when it was preserved with BAK.

These studies are representative of the detrimental side effects BAK can have on the corneal epithelium, yet multiuse bottles do require a preservative in order to prevent a much more serious bacterial infection. Therefore, what can be the solution?

With time, safer alternatives to BAK have been developed, such as polyquaternium-1, a cationic polymer that is 27-fold larger. With no hydrophobic region, or detergent properties, it cannot interact with mammalian cells [55]. Preservative-free, single-use eyedrops have also appeared on the market for select products; these have also been shown to cause less cell death compared to their BAK-preserved counter parts [14]. The existence of these alternatives begs the question, why are BAK-preserved versions still so prevalent in the consumer market when it has been previously shown to cause toxic complications? Its prevalence can be traced to its efficacy and early adoption as the antimicrobial of choice for the long-term storage of most forms of ophthalmic solutions including glaucoma treatment and artificial tears [56]. In some cases, the inclusion of a preservative that can increase the permeability of cells in the corneal epithelium is by design, allowing the penetration of the active ingredient to enter the anterior chamber [57]. Nevertheless, the state the ocular surface is left in can leave the cornea more vulnerable than it already is. In cases of OA, the use of anti-allergy drops is for the purpose of relieving surface-level itching localised to the epithelial immune pathways; in this use case, penetration into the anterior chamber is not required. Is it possible that the administration of these preserved eyedrops is cumulatively increasing risk factors for the development of corneal ectasia? In situations in which inflammation persists and eye rubbing is already a frequent occurrence, could weakening the corneal structure by causing mass cellular deterioration increase the likelihood of deformation? The answer to this question is pivotal and requires further research.

## 6. Corneal Ectasia

KC is a disease that has persisted through time with much conflict in the academic world, with many publications challenging its prevalence and aetiology. With advances in technology and analytical techniques, KC has seen a greater consensus to its classification as an inflammatory-mediated disease [58]. Despite its characteristic late-stage corneal deformation, KC is often difficult to diagnose in the early stages due to the inability of practitioners to visually distinguish a keratoconic cornea from a healthy one during routine examinations [59]. Recent advances in computer-aided diagnostic systems have proposed ways for early detection using tomography methods [60].

It has been found that the prevalence of KC can vary depending on geographical location. A study of keratoconus prevalence in the United States found that the frequency of KC in the US population is 1:1835 [61]. However, a study in the rural villages of Central India determined that the prevalence of KC was approximately 1:43 [62], and a population-based study of rural Iran found the prevalence of KC to be as high as 1:25 [63]. The initial signs of KC can manifest as early as adolescence and may progress into later stages over the course of multiple decades, affecting individuals for a majority of their lifespan [64]. The burdens of KC stem from complications associated with the protruding and thinning of the cornea. Individuals who have healthy corneas show rigid structures that have highly organised extracellular matrix collagen networks to allow for the passage and focussing of light waves. When integrity is compromised in cases of KC, the rigidity is lost, leaving the cornea susceptible to applied forces, such as mechanical eye rubbing. The curvature of the cornea is important in focusing light waves into the vitreous body and retinal neural layer to produce sharp imagery. Alteration to its spherical form, as seen in cases of KC, leads to irregular astigmatism, the shift of the focal point and, in turn, blurred vision that is very difficult to correct [20,59]. Studies have shown a decrease in corneal epithelial and stromal cell density with increased breaks in the Bowman’s layer in individuals with KC compared to healthy individuals [29,65,66]. From this, it is inferred that apoptotic corneal cell loss can be a causative factor in the pathogenesis of KC.

Genetics are one potential factor of KC, but there are many environmental factors that have been linked to the progression of KC. Despite having a history dating back 167 years, KC is associated with so many complications and potential causes that determining its specific aetiology has proven difficult; for a large portion of history, KC was documented as a non-inflammatory disorder.

The challenge for classifying KC as an inflammatory disease has been a historical lack of statistically significant expression of proinflammatory biomarkers present in studies conducted in the 20th century [7]. Studies conducted after the turn of the new century have shown more consistent results with a statistically significant expression of common inflammatory biomarkers, such as interleukin-6 (IL-6) and inflammation-associated proteinases such as matrix metalloproteinases (MMPs) in KC patients compared to control group [15,67,68], challenging the pre-existing classifications.

MMP-9 (gelatinase B) is a part of a group of zinc-dependant proteinases expressed by inflammatory mediating cells in cases of cell injury. During the body’s wound healing response, MMP-9 is upregulated to breakdown the extracellular matrix of damaged cells, allowing for the removal and replacement of affected cells within the human cornea [69]. With increased expression, however, MMP-9 can cause damage to otherwise healthy cell tissues, degrading their collagen networks and, by extension, their structural rigidity [19]. The increased presence of MMP-9 in tear samples has often been associated with more severe cases of KC rather than cases that could be considered mild or moderate [70]. Due to this, MMP-9 has been relentlessly investigated for its role in KC. Many studies have shown that MMP-9 is upregulated in allergic response pathways and is also present in high concentrations in cases of KC [7,26,68,71]. Additionally, Balasubramanian et al., 2013 [42] demonstrated that prolonged eye rubbing is often observed in those with progressed KC symptoms, increasing the presence of pro-inflammatory cytokines and multiple MMPs. With this, the researchers were able to establish a link between inflammation, eye rubbing and KC. The progression of the cornea to the characteristic conical shape has also been attributed to the repetitive stress applied via mechanical pressure from eye rubbing [7,58]. In a study focussed on detection methods for KC, non-invasive MMP-9 testing of tear film samples showed promise as a detection tool for KC progression risk in allergy-prone patients [71]. Nishtala et al., 2016 [26] demonstrated that the treatment of corneal epithelial cells with cyclosporine A, an immunosuppressant treatment method, reduced KC disease progression and decreased MMP-9 levels present in tear samples.

The past couple of decades have provided more evidence than ever before that inflammation plays a significant role in the progression of KC.

## 7. Conclusions

In conclusion, from the information gathered in this review, it seems clear that the rise of allergy prevalence in society and its links to KC through the expression of inflammatory proteins, atopic degradation or associated risk factors such as eye rubbing paint a troubling picture. The human eye is a complex mesh of collagen networks that are arranged in a very precise manner that if disturbed can affect vision. In the case of KC, vision can be impaired to a chronic and permanent degree if not intervened by novel treatments or corneal transplants. Commercial treatments for allergy exist to provide momentary alleviation of allergy symptoms and require continuous application after each exposure to the allergens. Increased levels of MMP-9 due to the localised ocular immune response, coupled with the increased levels of MMP-9 and mechanical pressure observed after prolonged eye rubbing, have been attributed to the progression of KC. Considering the well-documented impact that the previous literature has detailed, the use of BAK-preserved anti-allergy medication may be adding an additional risk factor for KC. A risk factor that is not necessary in the current day with antimicrobial alternatives and preservative-free options. A more in-depth understanding of the potential interactions topical anti-allergy treatments have in the context of corneal deformation is required in order to preserve patients’ vision in the future.

## Figures and Tables

**Table 1:** Common classes of commercially available eyedrops and their respective active ingredients, preservative options, recommended duration of treatment and their Australian scheduling classification.

Mechanism of Action	Active Ingredient	Preserved with	Duration of Treatment	Scheduling
Antihistamine	Levocabastine	Benzalkonium chloride	Indefinite	Schedule 2
	Pheniramine maleate	Benzalkonium chloride	Indefinite	Schedule 2
Mast cell stabiliser	Sodium cromoglycate	No preservative	>2 weeks	Schedule 2
	Lodoxamide	Benzalkonium chloride	>2 weeks	Schedule 2
Combination	Ketotifen fumarate	Benzalkonium chloride	Indefinite	Schedule 2
	Olopatadine	Benzalkonium chloride	Indefinite	Schedule 4
	Azelastine	Benzalkonium chloride	Indefinite	Schedule 2
Corticosteroid	Fluorometholone	Benzalkonium chloride	<2 weeks	Schedule 4
	Dexamethasone	Benzalkonium chloride	<2 weeks	Schedule 4

## Data Availability

Not applicable.
